# Online Conference “Chronic Viral Infections and Cancer, Openings for Vaccines and Cure” VIRCAN2024, Monitoring the Progress

**DOI:** 10.3390/vaccines13090940

**Published:** 2025-09-02

**Authors:** Liba Sokolovska, Juris Jansons, Franco M. Buonaguro, Maria Isaguliants

**Affiliations:** 1 Institute of Microbiology and Virology, Riga Stradins University, LV-1067 Riga, Latvia; 2 Latvian Biomedical Research and Study Centers, LV-1067 Riga, Latvia; 3 Molecular Biology and Viral Oncology Unit, Istituto Nazionale Tumori IRCCS Fondazione Pascale, 80131 Naples, Italy; 4 Department of Microbiology, Tumor and Cell Biology, Karolinska Institutet, 17177 Stockholm, Sweden

**Keywords:** vircan2024, virus, infection, oncovirus, vaccine

## Abstract

Chronic viral infections and virus-induced cancers have been actively studied for decades, with many significant advancements in basic science, disease cure, treatment, and prevention. Yet, today, these infections and pathologies remain major contributors to morbidity and mortality worldwide. The international online conference “VIRCAN2024: Chronic viral infections and cancer, openings for Vaccines and Cure” aimed to address the remaining issues, present the research carried out in this broad field, and prognose directions for its development. The conference covered oncogenicity mechanisms and new approaches in the development of treatments and vaccines. VIRCAN2024 was held on the platform of Riga Stradins University, Riga, Latvia. The conference was supported by the Latvian Science Council grant “Human papillomavirus genome associated correlates of disease progression and treatment response for cervical neoplasms and cancer”, and the scientific journal *Vaccines* (MDPI). This report summarizes the lectures and presentations given at the conference.

## 1. Online Conference “VIRCAN2024: Chronic Viral Infections and Cancer, Openings for Vaccines and Cure”

Chronic viral infections and virus-induced cancers remain major contributors to the global burden of infectious diseases and cancers, morbidity, and mortality of millions of people, as well as huge pressure on the national health care systems. Over 4% of the global population are living with chronic viral infections, such as HBV and HCV (304 million people) and HIV-1 (40.8 million) [1,2]. Nearly 10% of cancer cases worldwide are attributed to viral infections, with HPV and HBV having the highest age-standardized global incidence rates–18.5 and 3.9 per 100,000 person-years, respectively [3,4]. Even though science and medicine have made substantial progress in treating and preventing many viral infections, multiple issues remain to be addressed and resolved.

The lingering problems of viral persistence and oncogenicity were addressed by the international online conference “Chronic viral infections and cancer, openings for Vaccines and Cure” (VIRCAN2024) hosted by Riga Stradins University with the support from the Latvian Science Council grant “Human papillomavirus genome associated correlates of disease progression and treatment response for cervical neoplasms and cancer”, (https://www.rsu.lv/en/project/human-papillomavirus-genome-associated-correlates-disease-progression-and-treatment, accessed on 31 July 2025) and the scientific journal VACCINES (MDPI). Conference continues the tradition of the online and hybrid workshops and conferences organized by the University. The previous one “Vaccines and Vaccination During and Post Covid Pandemics” (VAC&VAC2022) was held online 7–9 December 2022 with materials, including abstract book, available on the conference web (https://www.rsu.lv/en/vac-vac-2022, accessed on 31 July 2025). Selected articles were published in the special issue of VACCINES “Pandemics-Born Revolution in the Preclinical and Clinical Trials of Microbial Vaccines” (https://www.mdpi.com/journal/vaccines/special_issues/FH7P87X1J4, accessed on 31 July 2025).

The VIRCAN2024 Conference gathered nearly 80 participants from 9 countries, including the Czech Republic, France, India, Italy, Latvia, Uganda, Ukraine, Sweden, and the USA. It covered the topics of viral infections which cause cancer, course and outcome of chronic viral infections, and innovations in the viral infection treatment, screening and prevention by vaccination. The conference lasted for two days and was divided into three focused sessions. The first session “Common and Specific Mechanisms of Viral Oncogenicity”, covered topics related to the mechanisms of viral oncogenicity. The second session “Approaches to Chronic Viral Infection and Cancer Cure” focused on the vaccines and treatment for cancer and chronic viral infections. The third session was devoted to one of the most notorious oncogenic viruses, namely high-risk human papillomaviruses (HPV)–“Human Papillomavirus, Chronic Infection and Associated Cancers, on the Way to Protection”. Conference participants with the status of early career researcher (35 years and younger) had the opportunity to participate in the “Early Career Researcher Contest”, with the winners being awarded monetary rewards, and two best presenters, with the vouchers for publication of their data in the special issue of the scientific journal “Vaccines” dedicated to the conference “**Chronic Viral Infections and Cancer: Openings for Vaccines and Cure**” (https://www.mdpi.com/journal/vaccines/special_issues/PD0W1PZ6L6, accessed on 31 July 2025).

Conference was opened by the lecture by Dr. **Elena Kashuba** (Kavetsky Institute of Experimental Pathology, Ukraine, and Karolinska Institutet, Sweden), “**DNA Tumor Viruses and Their Role in the Development of Epithelial Tumors**”. The lecture gave an overview of the malignant cell transformation, and DNA viruses involved in the development of tumors. The focus was on the herpesviruses such as Epstein-Barr virus (EBV), Kaposi sarcoma virus (HHV8), Herpes virus Saimiri (HVS), Marek’s disease virus (MDV), and human papillomaviruses. Dr. Kashuba highlighted the main cellular pathways (Rb and p53) involved in virus-induced cell transformation and the mechanisms of how viruses inactivate cell cycle regulatory pathways.

## 2. Common and Specific Mechanisms of Viral Oncogenicity

The first session of the conference hosted four plenary lectures and eight oral presentations, with four of them made by the early-career researchers. The session had three sections: [1] Molecular drivers of malignant transformation; [2] Metabolic signatures of chronic viral infections & cancer; [3] Immune response in chronic viral infections and cancer (Inflammation & Immune Evasion), each opened by the invited plenary lecture from the prominent researchers leading the corresponding field.

### 2.1. Molecular Drivers of Malignant Transformation

The section was chaired by Dr. Elena Kashuba and was opened by a plenary lecture presented by Dr **Maria Lina Tornesello** from the National Cancer Institute (Istituto Nazionale Tumori–IRCCS Fondazione Pascale), Naples, Italy, titled “**Mechanisms of Telomerase Reverse Transcriptase Reactivation in Cancer**”. Maria Lina Tornesello provided an overview of recent findings concerning telomerase and telomerase reverse transcriptase (TERT) promoter mutations in cancer, their application in diagnostics, and potential treatment development. Telomerase is a ribonucleoprotein complex consisting of several proteins, including TERT and the RNA template component (TERC). Telomerase activity is crucial to sustaining the unlimited proliferation of cancerous cells. High frequencies of mutations in TERT have been documented in numerous cancers and are thought to play a crucial role in the reactivation of telomerase. Additionally, TERT mutations are the most common mutations in cancer, even surpassing p53, and in some cancers, such as bladder cancer, gliomas, and thyroid cancer, the mutation rates exceed 50%. These mutations can create new transcriptional factor binding sites, leading to the expression of telomerase, where transcriptional factors are specific to certain tumor types [5]. Many oncoviruses have also been shown to activate TERT expression, either by directly integrating into the TERT gene locus, thereby causing its expression, or through viral oncoproteins such as E6 and E7 (HPV) and LMP1 (EBV), among others. Dr. Tornesello continued by highlighting TERT mutations present in genital cancers and head and neck cancers. Her group demonstrated that while more HPV-negative cancers harbored TERT mutations, a certain percentage of TERT mutation-positive cancers were also positive for HPV. They were able to demonstrate that TERT mutations cause a much higher telomerase activity when compared to HPV oncoproteins [5,6]. TERT mutations have been identified as early events in some tumor types and late events in others. In glioblastomas, melanoma, hepatocellular carcinomas (HCC), urothelial bladder cancers, and, as demonstrated by Dr. the Tornesello’s group in the National Cancer Institute, also penile cancers and conjunctival carcinomas, where TERT mutations appear as the early events of oncogenesis [7,8]. The talk highlighted that detailed characterization of TERT mutations is crucial in developing the diagnostic and therapeutic approaches based on the telomerase (TERT). Dr. Tornesello finalized the lecture with an overview of the therapeutic strategies targeting telomerases. She highlighted several approaches and drugs that have been created to either target the TERT promoter and the transcription process, or the telomerase enzymatic functions. One class of drugs was highlighted which acts as silencers and either block the TERT promoter, or stabilizes the Myc/Max or Wnt/Klf4 complexes. One drug Cantide, that targets the TERT mRNA through antisense oligonucleotides, is currently in clinical trials for hepatocellular carcinoma. Other affect the formation of telomeres, either by targeting the telomere site or by blocking the enzymatic activity of telomerase [9].

The next talk “**Role of Voltage Dependent Anion Channels in the HBV Life Cycle**” was given by **Pierre-Louis Schmit-Vergely** (University of Lyon, Université Claude-Bernard, Cancer Research Center of Lyon, Lyon, France), one of the Early-Career researchers The talk, albeit focused on the broader topic of HBV-related liver diseases, had a specific focus on one of the viral proteins, HBx. The HBx protein has been shown to interact with mitochondria by decreasing the membrane potential, increasing the levels of reactive oxygen species and Ca^2+^, promoting mitochondrial fission, and facilitating the perinuclear aggregation of mitochondria [10,11,12,13,14,15]. Of particular interest to the presenter was the reported interaction between HBx and mitochondrial outer membrane proteins, specifically voltage-dependent anion channels (VDAC) [16]. VDAC proteins are the most abundant proteins in the mitochondrial outer membrane, and they exist in three isoforms: VDAC1, VDAC2, and VDAC3. VDAC1 is the most abundant of the three and is responsible for multiple metabolite and ion exchanges. It also plays a role in apoptosis, where, upon pro-apoptotic stimuli, the proteins oligomerize, creating large pores that release other pro-apoptotic factors, such as cytochrome c, which leads to cell apoptosis [17]. Data from the presenter’s lab had indicated that HCC patients have elevated levels of VDAC1, and viral proteins interact with VDAC1 in the case of the hepatitis C virus [18]. Thus, by using a cell culture model with inducible expression of HBx, the presenter investigated the potential interaction between VDAC1 and HBx, as well as its role in mediating mitochondrial metabolism and apoptosis. Using VDAC1 immunoprecipitation, they demonstrated the association between VDAC1 and HBx. Additionally, through mitochondrial isolation, they confirmed the presence of HBx in the mitochondrial fraction. Unfortunately, they were able to demonstrate this only in the context of HBx overexpression. Additionally, a cytoprotective effect was observed in cells overexpressing HBx after treatment with various drugs known to cause apoptosis via VDAC1 oligomerization. Furthermore, using VDAC1 knock-out and knock-down cell culture models, they were able to show a reduction in HBx, leading to a hypothesis of a HBx-stabilizing role of VDAC1. Taking all these issues together, Pierre-Louis Schmit-Vergely highlighted the need for future research into this interaction to elucidate the role of mitochondria in the pathophysiology of HBV-related diseases.

Next talk titled “**HTLV-1C lung disease linked to p16 protein inhibition of efferocytosis in macaques**” given by Dr. **Sarkis Sarkis** (Animal Models and Retroviral Vaccines Section, Vaccine Branch, Center for Cancer Research, National Cancer Institute, Bethesda, MD, USA) covered studies of the human T-lymphotropic virus type 1 (HTLV-1) subtype C infectivity in macaque models. HTLV-1 is the first oncoretrovirus identified in humans. It has seven subtypes, among which subtype A is the most common one and is endemic in Japan, South America, Central Africa, the Caribbean islands, and some areas in the Middle East. HTLV-1 infects CD4 T cells and can be transmitted from mother to child via breastmilk, through sexual intercourse, or blood transfusions. A low percentage of those infected with HTLV-1 will develop one of the two major diseases associated with the virus-adult T-cell leukemia (ATL) or tropical spastic paraparesis/HTLV-1 associated myelopathy (TSP/HAM) [19]. However, a recent study demonstrated that HTLV-1 subtype C is endemic in central Australia, in Aboriginal people, with a prevalence of over 30%-the highest reported worldwide, and its clinical presentation also frequently includes lung diseases [20,21]. It is currently unknown what contributes to the increase in observed HTLV-1 subtype C-associated lung disease–whether it is the virus itself, the population genetics, or some other factors. The highest nucleotide divergence between subtypes A and C occurs at the 3′ end of the virus genome, in the *orf-I* region. Additionally, all the subtype C viruses that have been sequenced lack the translation initiation codon for *orf-I*. The *orf-I* encodes the p12 protein, which is cleaved to create the p8 protein. S. Sarkis has demonstrated that this protein is important in HTLV-1A infectivity and persistence by counteracting immune responses, as well as facilitating viral transmission [22]. Additionally, while studies in vitro have shown that p12 and p8 are dispensable, in macaques, their expression is crucial for ensuring HTLV-1A infectivity [23,24]. Thus, they aimed to investigate how the genetic differences between HTLV-1 A and C, specifically in the level of *orf-I*, could contribute to the pathogenicity of HTLV-1C. To achieve this, a chimeric HTLV-1A/C molecular clone was created in silico by cloning the entire *orf-I*, the overlapping *orfs II*, *III*, *IV*, and the 3′LTR of HTLV-1C derived from an infected human patient into the HTLV-1A backbone, and its infectivity was tested in human and macaque CD4^+^ T-cells in vitro and in macaques in vivo. The chimeric virus achieved similar infectivity rates both in vitro and in vivo, but differing inflammatory chemokine and cytokine profiles were found in both blood and bronchoalveolar lavage when the chimeric virus was compared to HTLV-1A–increased CCL13, IL-15, and IL-27, which are known to be important in lung lesion development, and decreased IL-18, IL-1β, and OLR1, which are known to be protective in lung disease. Additionally, histological investigation of lung tissues from macaques infected with the chimeric virus revealed lung lesions as well as immune cell infiltration (B cells, T cells, Monocytes, and macrophages) and the presence of viral proteins. They were able to demonstrate that despite the lack of translation initiation codon in the orf-I of HTLV-1C, subtype C orf-I (orf-IC) can be expressed in vitro and in vivo in the lungs of infected macaques by a doubly spliced mRNA juxtaposed with the first exon of rex, which provides its ATG in frame with orf-I and encodes the p16C protein (rex-orf-IC). Finally, they investigated how the p16C protein is involved in the development of lung lesions. They found that T-cells expressing p16C become resistant to engulfment by efferocytosis, a monocyte function that clears apoptotic cells and maintains tissue homeostasis. While these results are fascinating, more work will be done, by creating chimeric viruses lacking p16C.

Dr. **Irina Kholodnyuk** (Institute of Microbiology and Virology, Riga Stradiņš University, Riga, Latvia) gave the final presentation of the session titled “**EBV infection in treatment-naïve patients with chronic lymphocytic leukemia and mobility of the leukemic cells**”. Chronic lymphocytic leukemia (CLL) is the most common type of leukemia in adults and accounts for about 25% of new leukemia cases. The disease is highly heterogeneous, with some patients not needing any treatment at all, while about 50% of patients experience an aggressive form of disease, where early therapy initiation is crucial. In a subset of patients, CLL can transform into more aggressive forms of lymphoma, in most cases diffuse large B–cell lymphoma (DLBCL) and rarely Hodgkin’s lymphoma. EBV is a B-lymphotropic, oncogenic virus, closely associated with a number of lymphoproliferative diseases. While a relatively small portion of DLBCL is associated with EBV, Hodgkin’s lymphomas are associated with EBV in up to 70% of cases [25]. While EBV is not directly implicated in CLL, CLL can lead to immune dysregulation, which in turn can lead to increased infection risk. Additionally, three independent studies reported high EBV load in peripheral blood mononuclear cells of CLL patients, that were associated with worse overall survival and a more aggressive form of the disease [26,27,28]. Irina Kholodnyuk and group previously demonstrated that the EBV infection of B cells, isolated from blood of healthy donors, induces expression of the inflammatory chemokine receptors CCR1 and CCR2, but not CCR3 or CCR5 [29,30,31], thus the reported study aimed to examine whether the presence of EBV DNA and/or transcripts in PBMCs of treatment-naïve CLL patients is associated with the expression of CCR1 and/or CCR2 on leukemic (CD19+CD5+) cells. The study found that high EBV DNA load and co-expression of LMP1 and EBNA2 in PBMCs of treatment-naïve CLL patients were accompanied by an increase in the number of leukemic cells expressing CCR1, CCR2, and the negative prognostic marker CD38. Expression of CCR1 and/or CCR2 on CLL cells can promote migration of the leukemic cells into secondary lymphoid organs, which are enriched with chemokine receptor ligands, thus potentially contributing to progression of the disease. It could serve as a predictor of a more aggressive disease and a signal for the need for an earlier therapeutic intervention.

### 2.2. Metabolic Signatures of Chronic Viral Infections & Cancer

This second section was chaired by Dr. Juris Jansons (Latvian Biomedical Research and Study Centers, and Riga Stradins University, Riga, Latvia). It was opened by a plenary lecture “**Metabolic Changes in Viral Infections**” presented by Dr. **Ujjwal Neogi** (Systems Virology Lab, Department of Laboratory Medicine, Karolinska Institutet, Stockholm, Sweden). His lab has established a state-of-the-art interdisciplinary research platform integrating the computational biology, engineering, and clinical science to advance the understanding of metabolic rewiring achieved by RNA virus infection. The laboratory of Dr Neogi focuses on a panel of viruses, such as HIV-1, Crimean-Congo hemorrhagic fever virus, various coronaviruses, and also Dengue Virus. Dr. Neogi’s group is actively utilizing their workflow in HIV research, with a specific focus on metabolomics. The goal is to identify key components of the immune response essential for natural immune protection and disease severity, specifically, deciphering the mechanism of inflamm-aging in people living with HIV (PLHIV). The lecture by Dr Neogi gave an overview of the work of his group focused on the multi-omics analysis. Their workflow begins with samples from various patient cohorts acquired in the longitudinal or cross-sectional studies, alternatively in cell culture or in vivo animal models of viral infection and associated disease. These samples are then analyzed using “single omics”–transcriptomics, proteomics, metabolomics, lipidomics, and microbiome analyses. Finally, all sets of the omics data are combined, and depending on a specific context or disease, an integrative multi-omics analysis is performed to dissect the infection/disease scenario on the molecular level. This allows the group to identify how different omics “layers” interact and how these data could be used to create specific disease models, alternatively, specific health status models (infected, not-infected etc.). Finally, they perform mechanistic studies to validate their findings. Such integrative approach enables personalized medicine strategies, which take in individual metabolic and immune variations, promising advances in combating RNA virus infections and improving health outcomes [32]. Over the year, thanks to their multi-omics approach and ability to model and contextualize their results, they have been able to move from exploratory studies in various cohorts to identifying targets, validating them, and even proceeding to preclinical studies [33,34].

The next presentation “**Viral replication capacity influences HIV-1-induced metabolic and cytokine reprogramming of T Cells**”, was given by **Omolara Baiyegunhi** (Africa Health Research Institute, Durban, South Africa), one of the Early Career Researchers. Human immunodeficiency virus-1 (HIV-1) replicative capacity (RC) is thought to be important in several aspects of the infection. Firstly, it is thought to be one of the features of founder viruses–a small population of viruses in the blood that establishes infections in a new host [35,36]. Secondly, studies have demonstrated that high RC correlates with higher viral loads and a faster disease progression [37]. Omolara Baiyegunhi aimed to investigate how viruses with varying RC impact viral transmission and disease progression. They hypothesized that viral RC could affect inflammatory cytokine production, T cell metabolism, and cell-to-cell spread. To investigate this, they generated chimeric viruses by inserting patient-derived gag-protease regions from HIV-1 subtypes B and C (with high and low RC) into the NL4-3 backbone. Overall, they saw that the subtype B viruses had a higher RC than subtype C. Furthermore, they observed that the low RC subtype C viruses induced higher levels of several cytokines, such as TNF-α and IL-8, among others. Further cytokine analysis revealed associations between cytokine levels and viral RC, where IL-8, TNF-α, and IL-3 negatively correlated with virus RC, while IL-7, PDGF, and MCP-1 correlated positively. Next, they analyzed the RC effect on HIV cell-to-cell spread using in vitro T cell cultures and flow cytometric analysis. Cell-to-cell spread was least efficient in subtype C, with a low RC, thus implying that higher RC is associated with more efficient cell-to-cell spread. Metabolic function analysis revealed that higher RC was associated with higher glutamine and glucose consumption. Additionally, low replicative viruses were associated with higher mitochondrial mass but lower membrane depolarization. Taking all of this together, they were able to demonstrate that high RC HIV-1 strains trigger unique cellular responses, altering metabolism and cytokine profiles to accelerate disease progression.

The final talk of the section continued on the topic of HIV. Dr. **Alejandra Escós** (Division of Clinical Microbiology, Department of Laboratory Medicine, Karolinska Institutet, Stockholm, Sweden) presented the results of a study titled “**Disrupted alpha-ketoglutarate homeostasis trained monocyte-derived macrophages towards M2-like phenotype increase HIV infectivity in treated infection**”. Even though antiretroviral treatments have substantially increased the survival rate of people living with HIV (PWH), they still have a number of challenges, such as healthy aging and eradication of the infection reservoir. PWH experience accelerated aging, as well as the effects of residual inflammation and drug toxicity, since treatment has to be sustained indefinitely. Infected cell reservoirs represent just a couple of cells, usually CD4 T cells, but also cells of the myeloid lineage and microglia cells. Dr. Escós is especially interested in the myeloid lineage cells–macrophages. They are classically classified into the inflammatory M1 and anti-inflammatory M2 macrophages, but they are able to shift between these two states. The working hypothesis of the talk is that metabolic reprogramming and altered chemokine signaling in PWH on long-term antiretroviral therapy affect monocyte transport and polarization due to ongoing inflammation. Using a multi-omics approach (RNAseq, bulk and single-cell proteomics) in macrophages from PWH, they aimed to identify the mechanism of impaired monocyte/macrophage function in PWH. Single-cell proteomic analysis revealed that PWH had a hyperactive immunometabolism, with various metabolic pathways (glycolysis, pentose phosphate pathway, and others) being involved. This is crucial as viruses upon infection try to hijack their host’s metabolic pathways by activating the Krebs cycle, creating an imbalance in the redox and energy metabolism, as well as using up substrates of various pathways for viral particle production [38]. Using a model that integrates both transcriptomic and metabolomic data, they were able to demonstrate that alpha-ketoglutarate is crucial in the altered metabolism observed in PWH macrophages. Additionally, they confirmed these findings in plasma metabolomic data from 400 patients from four different cohorts and demonstrated that PWH sera had high levels of alpha-ketoglutarate. Further analyses revealed that PWH macrophages were polarized towards an M2-like state. The PWH macrophages were also shown to have high expression of CCR5–the cell entry receptor for HIV, and further experiments demonstrated that donor macrophages exposed to PWH sera polarized to a more M2-like state and again had an increased CCR5 expression. Finally, they demonstrated in vitro that donor macrophages treated with alpha-ketoglutarate had a higher HIV infectivity rate. In conclusion, using systems biology approaches the study demonstrated impaired macrophage polarization due to metabolic training, which leads to a low-grade nonclassical inflammatory environment in PWH.

### 2.3. Immune Response in Chronic Viral Infections and Cancer (Inflammation & Immune Evasion)

The third section of the conference was chaired by Dr. Irina Kholodnyuk (Riga Stradins University) and was opened by the plenary lecture from tenure “**Immune Response in SARS-CoV-2 Infection, Inflammation, and Cancer Immune Evasion and Tumor Progression**” presented by Prof. **Dace Pjanova** (Institute of Microbiology and Virology, Riga Stradins University, Riga, Latvia). The plenary lecture covered how the COVID-19 pandemic impacted those already suffering from cancer–their care, vulnerability to COVID-19, and how SARS-CoV-2 itself could be linked with cancer. Firstly, Prof. Pjanova reported the results of her group, which demonstrated that the COVID-19 pandemic had disrupted the routine cancer care, such as delayed routine screening, leading to later-stage diagnosis. Their results on melanoma patients demonstrated that during the pandemic, especially in 2020, more late-stage melanomas were diagnosed. Next, the talk highlighted how the development of COVID-19 in cancer patients significantly impacted several key health metrics. For example, all cause of mortality in cancer patients with COVID-19 was demonstrated to be 14%, while in those without COVID-19, it was reported as only 2%. And hospital admissions of cancer patients with COVID-19 were 64%, while those without were only 14%, showing how SARS-CoV-2 affects an already vulnerable population [39]. Further, the lecture shifted towards the link between inflammation, cancer, and COVID-19. Inflammatory processes are involved in all stages of tumorigenesis, with several inflammatory pathways, such as NFκB, Stat3, and cytokines, such as IL-1, IL-6, and TNFα, playing key roles [40]. Some of the key cytokines implicated in tumor progression are elevated in COVID-19, during the “cytokine storm”, thus leading researchers to believe that the created inflammatory environment could be tumor development-promoting. Additionally, SARS-CoV-2 Spike proteins have been shown to persist in patient blood after the acute phase of the infection [41]. In vitro studies have shown that the presence of the SARS-CoV-2 spike protein induces the production of several cancer and cell proliferation-associated cytokines and signalling pathways, but these responses differ depending on cell types [42,43,44]. Additionally, a study done in triple-negative breast cancer cells expressing increased levels of ACE2, the SARS-CoV-2 M protein induced a more aggressive behavior of the cells–increased proliferation, activation of the NFκB pathway, upregulation of epithelial-mesenchymal transition-associated genes, and increased production of inflammatory cytokines [45]. Future research must elucidate the molecular pathways involved and assess the long-term oncogenic risks associated with SARS-CoV-2 infections, guiding strategies to mitigate these risks.

Continuing the topic of COVID-19 association with oncogenesis, one of the Early Career Researchers, **Ganna Monchakivska** (RE Kavetsky Institute of Experimental Pathology, Oncology and Radiobiology of the National Academy of Sciences of Ukraine, Kyiv, Ukraine) gave a short presentation titled “**Influence of the COVID-19 disease on the course of Chronic lymphocytic leukemia**”. This presentation addressed some of the gaps in knowledge regarding how COVID-19 influences the course of CLL by analyzing the associative relationships between the immunophenotypic characteristics of malignant B-cells, the immune response of the patients, and concomitant coronavirus infection. Samples from CLL patients who had and hadn’t been infected with SARS-CoV-2, as well as samples from patients who had been vaccinated, were used to determine levels of cytokines and expression levels of immune signaling pathways, as well as for flow cytometric analyses. The study showed that CLL-COVID-19 patients had decreased levels of IL-10. In CLL-COVID-19 and/or COVID-19-vaccinated patients, increased levels of IL1β, IL6, IL8, IL17A, and GFS1 positively correlated with the relative survival. Ganna Monchakivska is continuing to explore this topic with a deeper investigation of various disease (both CLL and COVID-19) expression patterns.

Another Early Career Researcher **Enkhtuul Batbold** (Lyon Cancer Research Center, and University of Lyon, Université Claude-Bernard, Lyon, France) switched to a completely different virus, the hepatitis delta virus (HDV) giving a presentation titled “**CCL5 is upregulated and secreted by HDV independently of the IFN response**”. Liver cancer is the sixth most common cancer and the third most common cause of cancer-related deaths worldwide. It is predicted that the number of new cases of liver cancer per year will increase by 55% by 2040. The leading causes of liver cancer are the hepatitis viruses. Among them, HBV and HCV are considered oncogenic viruses. However, the role of HDV in hepatocarcinogenesis is still unknown, yet globally, 12 million people are living with a chronic HDV infection [46,47]. The HDV virus is the smallest known human virus, and its infection is totally dependent on HBV, as it needs its envelope to propagate; therefore, HDV infections occur only in the context of HBV infections. HDV+HBV is considered the most severe and aggressive form of viral hepatitis, yet the pathobiology is ill-studied due to the in vitro infection systems being extremely technically challenging [48]. Epidemiological data have revealed that Mongolia has the highest HDV prevalence (60.1%) worldwide [49], and transcriptional studies in this population revealed distinct inflammatory profiles [50], and later studies refining the inflammation-based classification of HCCs revealed a characteristic of increased IFN signalling as well as levels of CLL4 and 5 [51], which was the main focus of the reported study. The reported study found that HepaRG cells infected with either HDV alone or co-infected with HBV and HDV exhibited distinct transcriptional profiles compared to non-infected and HBV-infected cells. Pathway analyses highlighted marked differences, with a prominent role for interferon IFN signaling pathways. Further analyses indicated that HDV-induced molecular and metabolic alterations in hepatocytes are primarily mediated by IFN signaling, rather than by direct viral activity. Notably, a specific subpopulation of mono-HDV-infected cells demonstrated significant upregulation of CCL5, independent of IFN signaling. Moreover, CCL5 protein was detected in the culture supernatant of both HDV-infected HepaRG cells and primary human hepatocytes. This finding is potentially relevant to liver disease pathogenesis, as CCL5 is expressed across diverse myeloid and lymphoid cell types within the hepatic microenvironment and has been implicated in the progression of chronic liver diseases, including HCC. Collectively, the reported study uncovered a previously unrecognized upregulation and secretion of the chemokine CCL5 as a direct consequence of HDV mono-infection, potentially contributing to the pronounced inflammatory phenotype observed in HDV-infected livers and the accelerated progression toward fibrosis and HCC.

The next speaker Dr **Larysa Kovalevska** (RE Kavetsky Institute of Experimental Pathology, Oncology and Radiobiology of National Academy of Sciences of Ukraine, Kyiv, Ukraine) returned to the topic of CLL with presentation titled “**Identification of the “missing” transcription factors in Chronic leukocytic leukemia B-cells**”. This study aimed to shed light on the expression patterns of various transcriptional factors in B cells of CLL patients. Firstly, they reported the increased expression of POU2ATF1, JUNB, NFATC1, JUN, RELB, and FOS in CLL cells, as well as a reduced expression of C-MYC, HIF1A, ID1, and NFκB1, consistent with the nature of CLL cells, which practically do not proliferate. Immunofluorescent microscopy analysis of CLL cells revealed very low or even absent expression of SMAD3 and 4. They also analyzed the expression of the STAT family transcription factors and found that STAT4 is extremely reduced in CLL cells. In CCL cells, microscopy revealed very low levels of STAT5A and B, and its phosphorylated isoform was mainly localized in the cytoplasm of the cell, as opposed to the nuclear localization in healthy donor cells. The reported results indicated changes in several immune signalling pathways such as TGFβ-SMAD2/3 and IL-2-STAT2/5 (JAK-STAT5) in CLL patients.

## 3. Approaches to Chronic Viral Infection and Cancer Cure

The second session of the conference had one plenary lecture and four oral presentations and was chaired by Prof. Franco Maria Buonaguro (National Cancer Institute, Naples, Italy). The session was opened by the plenary lecture “**HBV Vaccination–Success and Barriers to Achieve Eradication**” given jointly by **Drs Isabelle Chemin** and **Boun Kim Tan** (Center for Research on Cancer, Lyon, France). The lecture highlighted the importance of the major global health issue of chronic HBV infection and the steps and strategies that need to be either improved or implemented to decrease the number of new infections, HCC-associated deaths, and achieve chronic viral hepatitis elimination. Chronic HBV infection is the leading cause of HCC worldwide, especially in Sub-Saharan Africa and Southeast Asia, with other regions having portions of either alcohol or HCV-related HCC cases [52]. Since HBV infection is largely asymptomatic, the diagnosis gets delayed, decreasing access to timely treatment and increasing the risk of HCC development. In high-income countries, HBV is more prevalent in high-risk populations, while in highly endemic areas, HBV is mostly acquired during birth or through horizontal transmission. Strategies to eliminate chronic viral hepatitis are based on immunization, early diagnosis, and treatment. Firstly, prompt vaccination is the most cost-effective way to prevent new HBV infections, which is essential in infants, especially in those born in endemic areas. Secondly, diagnosis and treatment-based HBV elimination strategies are based on access to affordable diagnostic tools, access to antiviral therapy, removal of stigma, creation of public health campaigns, improvement of diagnosis and treatment guidelines, and the development of curative therapies [53]. The last part of the lecture was devoted to the challenges of developing a cure. The lecture highlighted that to achieve this, both antiviral and immunotherapy approaches need to be combined, and research into the viral life cycle and pathophysiology needs to be continued to discover biomarkers for early “cure” prediction [54].

Next speaker, Dr. **Ratna Ray** (Department of Pathology, Saint Louis University, St. Louis, MO, USA) moved from immunization and viral vaccines to treatment with a talk titled “**Bitter Melon’s Sweet Promise: Role in Oral Cancer Therapy**”. Bitter melon (*Momordica charantia*) is a fruit often used in folk medicine as a remedy for diabetes, which is known to have beneficial effects on glucose metabolism and hepatic lipids. Dr. Ratna Ray’s group has extensively studied this plant and it’s active ingredient momordicine-I, and its potential use in the therapy of oral squamous cell carcinomas (OSCC). One of their first studies on bitter melon and OSCC demonstrated that bitter melon extract (BME) in drinking water reduced the formation of cancerous lesions. Further transcriptome analysis revealed that BME-treated cancers had lower levels of several markers present in OSCC development. Additionally, in vitro studies revealed that the BME has a positive immunomodulatory effect on T and NK cells, promoting their tumor-killing capabilities [55,56,57]. Following these encouraging results, they identified the active component of BME-momordicine-I, and further studies demonstrated that momordicine-I inhibited head and neck cancer cell growth and the protein c-Met and its downstream signalling, which is associated with head and neck cancer progression and metastasis [58]. Their latest work concerns the effect of momordicine-I on metabolic pathways in oral cancer and the tumor microenvironment (TME). They were able to demonstrate that momordicine-I induces tumor-relevant metabolic changes, such as decreased expression of glycolytic genes, inhibited lactate production, reduced Krebs cycle metabolites, and inhibited expression of lipogenic enzymes. Also, momordicine-I induced autophagy, disrupted mitochondrial function in oral cancer cells, and reduced tumor volume. In the TME, momordicine-I treatment caused a distinct transcriptomic signature, which involved immunomodulation that disrupted the tumor-promoting M2 tumor-associated macrophages and B-cell populations, thus restoring effective immunosurveillance in the TME. Together, their findings open new avenues for research and development of targeted therapies for oral cancer.

Continuing on the topic of oral cancers, Dr. **Subhayan Sur** (Cancer and Translational Research Centre, Dr. D.Y. Patil Biotechnology and Bioinformatics Institute, Dr. D. Y. Patil Vidyapeeth (DPU), Tathawade, India) gave a talk titled “**Exploring the Role of Long Non-Coding RNA in Oral Squamous Cell Carcinoma Development and Therapy**”. Long non-coding RNAs (lncRNAs) are RNA transcripts that lack protein-coding capacity, but interact with miRNA, mRNAs, DNA, or proteins to regulate a wide variety of cellular processes. Recent studies have indicated that lncRNAs are often abnormally expressed in various malignancies and are crucial for tumor growth and survival, making them promising candidates for cancer diagnosis and therapy [59,60]. Unfortunately, the role of lncRNAs in OSCC remains underexplored; thus, Dr. Sur aimed to investigate the role of lncRNAs in OSCC using patient samples. Through transcriptomic data, they were able to show that several biological processes were differentially modulated in OSCC patient samples, such as metabolic processes, cell communication, and cell proliferation, among others. Additionally, among the differentially expressed genes, they found that a novel lncRNA–EGFR long non-coding downstream RNA (ELDR), which was significantly upregulated in OSCC samples. ELDR is localized closely to the EGFR gene, and EGFR has been shown to be highly upregulated in several cancers [61]. Additionally, ELDR was shown to be associated with a higher grade of OSCC, to increase cancer cell proliferation by enhancing the expression of EFGR and cyclin E1 [62]. Further experiments revealed that normal oral keratinocytes with overexpressed ELDR gain a selective growth advantage for neoplastic transformation [63], and intratumoral administration of EDLR siRNA in OSCC-model mice regressed OSCC growth. To conclude, the lncRNA ELDR may serve as a driver gene in OSCC, and targeting this gene holds potential therapeutic significance.

Dr. **Anna Zajakina** (Latvian Biomedical Research and Study Centre, Riga, Latvia) continued with presentation “**Viral Vectors and Photosensitive Drugs as Immunomodulators in Cancer Treatment**”. The talk focused on alphavirus-based viral vectors and combining them with photodynamic therapy to target TME, especially in the case of “cold” TME, where immunotherapeutic drugs have low efficiency. One of the approaches to reprogramming immunosuppressive (“cold”) TME uses the delivery of transgenes, encoding cytokines, chemokines, or other immunostimulating molecules, which can help promote the transition to an immunostimulating (“hot”) TME [64]. Dr. Zajakinas’ group focuses on alphavirus-mediated delivery of cytokines that target both the immune cells and the tumor stroma. Their previous results using vectors carrying CLP, IL-2, and IFN-γ, showed promising results [65,66], but complete clearance of the tumor was not achieved; thus, they aimed to combine the alphavirus vectors with other forms of treatment, which cause immunogenic cancer cell death with a focus on macrophage activation. They turned to photodynamic therapy (PDT)–an approach that uses photosensitizers, which, upon activation by a specific wavelength of light, produce reactive oxygen species (ROS) that selectively destroy targeted cells or tissues by inducing immunogenic and ROS-mediated cell death, which in turn releases danger associated molecular patterns (DAMPs) and cytokine and further activates immune cells [67,68]. In the reported study, Dr. Zajakina and her group investigated the therapeutic potential of combining Aplhavirus vectors carrying IFN-γ with PDT using chlorin e6 in both 2D and 3D in vitro models, co-culturing cancer cells with macrophages. They demonstrated that combined therapy had a synergistic effect, with greater efficacy observed when viral vector treatment preceded PDT. Additionally, they observed enhanced phagocytosis of treated cancer cells by both M1 and M2 phenotypes in co-culture. Importantly, 3D-culture conditions maintained macrophage polarization stability post-treatment.

The final talk of the session–“Vaccine and SAMT-247 continuous released by intravaginal ring (IVR) decreased risk of SIV acquisition in macaque”, was presented by Dr. Mohammad Arif Rahman (Animal Models and Retroviral Vaccines Section, National Cancer Institute, Bethesda, MD, USA). For years, researchers around the world have tried to develop HIV vaccines. Among the many clinical trials conducted, only one–RV144 (ALVAC) showed a marked vaccine efficacy of 31.2% [69] with later studies in macaque models corroborating these findings [70,71]. Continuing to investigate macaque models, it was revealed that high anti-V1 antibodies were associated with an increased likelihood of acquiring an infection; thus, researchers modified the vaccine to exclude the V1 antigen and could observe an even higher vaccine efficacy in macaques, reaching as high as 65% in females [72,73]. To increase the efficacy even more, they tried to combine the vaccine with various adjuvants or microbicides. SAMT-247 is a microbicide and a potent HIV-1 nucleocapsid inhibitor, and by using it as part of a vaginal gel, they were able to demonstrate significant protection against vaginal simian immunodeficiency virus (SIV) challenge. Next, they decided to combine both the modified vaccine and the SAMT-247 vaginal gel and demonstrated a 92.7% vaccine efficacy [73,74]. Since the use of vaginal gel is not user-friendly, they developed an intravaginal ring (IVR) that continuously releases SAMT-247 and combined it with the modified vaccine. Remarkably, the combination of the vaccine with SAMT-247 IVR led to an 84.2% decrease in virus acquisition, and the SAMT-247 IVR alone also significantly reduced the risk of SIV acquisition. Additionally, they demonstrated that SAMT-247 not only acts as an antiviral but also modulates the immune system by enhancing NK cell activity, which in turn boosts antibody-dependent cellular cytotoxicity activity, further correlating with a decreased risk of SIV infection. In conclusion, SAMT-247 increased the protective immune responses generated by vaccine, leading to a reduced risk of SIV infection. Additionally, controlled release of SAMT-247 by intravaginal ring, combined with the a vaccination regimen, might result in an efficacious preventative strategy to lessen the global burden of HIV.

## 4. Human Papillomavirus, Chronic Infection & Associated Cancers, on the Way to Protection

The third and final session of the conference had four plenary lectures and ten oral presentations, with six of them by the early-career researchers. The session was divided into four sections: (1) Epidemiology & Risk Factors; (2) Mechanisms of HPV-associated Carcinogenesis; (3) HPV Infection & Cancer Surveys; (4) Prophylactic and Therapeutic HPV Vaccines & their Implementation.

### 4.1. Epidemiology & Risk Factors

The first section was chaired by Dr. Edward Kumakech (Lira University, Lira Uganda) and was opened by a plenary lecture from Prof. **Anda Kivite-Urtane** (Institute of Public Health, Riga Stradins University, Riga, Latvia) “**Epidemiology of Infection with High Risk HPVs, Risk Factors in Different Population Groups**”. The talk focused on HPV and HPV-associated cancer prevalence, groups at risk of HPV, and presented data from a recent study exploring the situation in Latvia–a country with one of the highest cervical cancer incidence rates in Europe [75]. Understanding the risk and associated factors of HR-HPV infections in different populations and societal groups facilitates the development and implementation of targeted, and therefore more effective, primary, secondary, and tertiary prevention measures and can help mitigate the global health burden that HPV poses. Certain groups have been identified to be at a higher risk of HPV, such as PWH, immunocompromised individuals, people undergoing immunosuppressive treatment, and people with concomitant sexually transmitted infections. When it comes to cancer development, while HPV is the main trigger in several cases, other factors that people can be exposed to also increase the risk of HPV-related cancer development, such as smoking, high parity, long-term hormonal contraception use, other infections, immune dysregulation, and even dietary deficiencies [76]. The talk finished by reporting the results of a study conducted by Prof. Kivite-Urtane’s group, where they analyzed HR-HPV prevalence and associated factors in Latvian women who visited either a colposcopy clinic or a general practitioner [77]. The study found that the HR-HPV prevalence in the general population of Latvia was 11%, in line with the European average [76], while in the colposcopy group, the prevalence reached 66%. Interestingly, the prevalence of HR-HPV decreased with age but showed a second peak among women aged 60 and older, possibly due to immune response changes in advanced age. The study demonstrated that in Latvia, factors associated with a positive HR-HPV status included being single, divorced, or widowed (as opposed to being married or cohabiting), having three or more lifetime sexual partners in the general population, and being of Latvian ethnicity (as opposed to other ethnicities) and being a smoker in the colposcopy group.

The next three presentations were from the Early-career researchers. Firstly, **Viola Daniela Kiselova** (Institute of Microbiology and Virology, Riga Stradins University, Riga, Latvia) presented her study entitled “**Sociodemographic and general health factor association with high-risk human papilloma virus infection in a prospective study of females residing in Riga, Latvia**”. She reported the data from a study aimed to identify factors associated with HR-HPV infection in healthy females residing in Latvia, made within the project of the Latvian Science Council “Human papillomavirus genome associated correlates of disease progression and treatment response for cervical neoplasms and cancer” (LZP-2021/1-0484). The study utilized data from a questionnaire covering sociodemographic data and general health information, clinical examinations, anamnesis, and HR-HPV genotyping. Firstly, 19 (22.2%) women of the study population were shown to be HR-HPV-positive. In contrast to earlier findings, HR-HPV positivity was not associated with sociodemographic factors, and there was no significant difference in HR-HPV prevalence between those who participated in cervical screening and those who did not. However, HR-HPV infection was significantly more prevalent among women who used contraception methods other than condoms or IUDs and in those with limited knowledge of HPV vaccination. Interestingly, HR-HPV infection was significantly more prevalent in females who reported no health-disturbing problems. Additionally, participants who had undergone colposcopy or had cervical health issues showed higher HR-HPV positivity. No associations were found between HR-HPV positivity and pain-related symptoms (backaches, etc.) and menstrual abnormalities. However, HR-HPV-positive women were more likely to have abnormal cervical tissue findings. In conclusion, the reported study found that HR-HPV infection was significantly associated with self-reported health status, ineffective contraceptive use, history of colposcopy, and cervical tissue abnormalities, as well as a lack of knowledge about HPV vaccination. These results highlighted the urgent need for increased awareness of HPV, HPV prevention, and HPV-related disease prevention.

**Justine Grundmane** (University of Latvia, and Gulbis laboratory, Riga, Latvia) switched to the HR-HPV genotype prevalence in Latvia in a talk titled “**Human Papilloma Virus Genotype Distribution Among Young Women in Latvia with Pathological Findings in Cervical Cytology**”. The presentation reported the results of a study investigating HR-HPV prevalence in women under the age of 30 who had pathological findings in cervical cytology. The study focused on this particular group due to the cervical cancer screening algorithm employed in Latvia. Cervical cancer screening in women aged 25 to 29 is primarily screened using cervical cytology, while after 30 years of age, HR-HPV genotyping is used as the primary screening technique [78]. The study included samples from 32 women who had pathological findings in cervical cytology (ASC-US, LSIL, HSIL). Most women tested HR-HPV-positive (24/32), with equal distribution of single and coinfections. The most prevalent HR-HPV genotype was HPV 66 (25%), HPV 16 and HPV 56 (both 21.88%), HPV 51 (18.75%), HPV 39 (12.5%), HPV 58 (9.32%), HPV 31, HPV 33, HPV 52 (each 6.25%), HPV 18, HPV 45, HPV 59, HPV 68 (each 3.13%), while the only genotype not detected was HPV35. The reported study revealed very interesting prevalence trends, with many of the most prevalent genotypes not being covered by the vaccines currently being used in the Latvian HPV immunization campaigns.

The final early-career researcher presentation of this section continued on the topic of HPV genotype prevalence with a talk titled “**Shift in High-Risk HPV Genotypes in Cervical Lesions in Latvia**” by **Liba Sokolovska** (Institute of Microbiology and Virology, Riga Stradins University). The study aimed to shed some light on several relatively unexplored issues in Latvia by analyzing cervical tissue samples from cervical cancer and dysplasia obtained from 2016 to 2024. Firstly, most HR-HPV prevalence studies in Latvia have been done in cervicovaginal smear samples from women undergoing routine cervical cancer screening, with only one study from 2004 collecting samples from women with cervical disease [79]. Secondly, researchers have proposed that the introduction of HPV vaccination (in many countries starting with the bivalent Cervarix vaccine targeting HPV16 and 18 can cause other non-vaccine genotypes to increase in prevalence [80,81], and this has not been analyzed in Latvia to date. The study was supported by the project of the Latvian Science Council “Human papillomavirus genome associated correlates of disease progression and treatment response for cervical neoplasms and cancer” (LZP-2021/1-0484). The study included tissues from 77 cervical cancer patients and 51 dysplasia patients and demonstrated that HPV16 was the most common genotype, followed by HPV33. HPV56, HPV52, and HPV45 were found only in cancer, while HPV31 and HPV66 were found only in dysplasia. Moreover, an observable decrease was seen in HPV16 prevalence and a significant decrease in HPV18 prevalence. Of note, both HPV33 and HPV45 increased in prevalence between 2016 and 2024. The study demonstrated that the prevalence of HPV16 and 18 seems to be decreasing, which is in line with the fact that they are the ones targeted by vaccines the longest. Still, other genotypes (HPV33 and 45), which were introduced only in the Gardasil9 vaccine, seem to be on the rise, highlighting the continued need for health education, vaccine awareness, and continued monitoring of circulating HR-HPV genotypes.

### 4.2. Mechanisms of HPV-Associated Carcinogenesis

The second section in the HPV session was chaired by Dr. Maria Isaguliants (Riga Stradins University, and Karolinska Institutet, Stockholm, Sweden) and was opened by the plenary lecture from Prof. **Franco Maria Buonaguro** (Istituto Nazionale Tumori–IRCCS Fondazione Pascale, Naples, Italy) “**HPV-Driven Mucosal Carcinogenesis**”. The plenary lecture covered the steps and factors involved in HPV-associated cancer development. The discovery of HPV and its link to carcinogenesis has been a long and arduous journey, which has resulted in an immeasurable impact on global health via HPV screening and vaccination. Much like other oncoviruses, HPV encodes oncoproteins that orchestrate processes incredibly important in malignancy establishment. The main HPV oncoprotein E6 and E7 interaction has been well described and mainly revolves around the inhibition and degradation of several tumor suppressor proteins [82,83]. While in the case of several cancers, like cervical and anal, most cases are attributable to HPV, several other factors are also closely linked to cancer development. Alongside HPV oncoproteins, other HPV-related processes are important in carcinogenesis, like the immune response against the infection, viral persistence in the tissues, and viral genome integration, transactivation of HPV by other viruses (like HIV), as well as the cells inherent genetic instability (mutations in p53, telomerase promoter etc.)

The next two topics were presented by the Early-Career Researchers. The first titled “**HPV16 up-regulates the expression of RNA-binding protein Sam68 in head and neck cancers**” was given by **Andrea Cerasuolo** (Molecular Biology and Viral Oncology Unit, Istituto Nazionale Tumori-IRCCS Fondazione G. Pascale, Napoli, Italy). Only a portion of head and neck squamous cell carcinomas (HNSCC) are attributable to HPV, and continued research has identified that HPV-related HNSCC have distinct clinical and molecular characteristics–they are more sensitive to radiation therapy, have a better survival, and exhibit specific mutation patterns [84,85]. These findings highlight the need to distinguish HNSCC patients in the most sensitive matter possible. Unfortunately, classical PCR analyses detecting HPVs may not be the best choice as they are not able to distinguish between transient and transforming infections; thus, more sensitive markers of active HPV infection are necessary. One of these markers could be the isoform of the E6 mRNA–E6*I, which has already been shown to be increased in high-grade lesions and cancer [86,87]. HPV transcripts isoforms are created through splicing, which is regulated by the major spliceosome complex and RNA-binding proteins. Different studies investigated the role of splicing factors and RNA-binding proteins (RBP), such as Sam68, in the production of the E6*I isoform [88,89]; thus, the reported study aimed to analyze the expression of these splicing factors in HPV-related and unrelated HNSCC and their possible interplay with HPV oncoproteins. The expression of SRSF3, BRM, and SAM68 was significantly higher in HPV-related than HPV-negative HNSCCs. Specifically, SAM68 levels were shown to correlate with E6*I expression. Accordingly, the SRSF3, BRM, and SAM68 were found significantly up-regulated in HPV-related HNSCC. Taking these results together with the known association of the E6*I isoform with malignant lesions, suggests new innovative therapeutic approaches could be based on Sam68 inhibitors.

The second “**Complete genome analysis of the human papillomavirus type 16 (HPV16) isolates from cervical carcinomas in Latvia**” was given by **Nikita Zrelovs** (Scientific Laboratory of Molecular Genetics, Riga Stradins University, and Biomedical Research and Study Centers, Riga, Latvia). The presentation summarized data from a study aiming to update our knowledge on the variability of HPV16 circulating in Latvia. Even though HR-HPVs are quite conservative and genetically stable, they can mutate and, with mutations, influence pathogenicity and anti-viral immune response. Such variability could shape the course of HPV-associated lesions and cancer and may affect the efficacy of anti-HPV vaccines [90]. The study used 24 DNA samples derived from pathological cervical tissues sampled between 2012 and 2023, shown to harbor high HPV16 loads–either as a mono-infection with HPV16 (*n* = 20) or coinfected with HPV33, 39, and 56 (*n* = 4). In 16 samples, the whole 7906 bp long HPV16 genome could be assembled, but only for one HPV33. Assembly could not be achieved in the other samples due to high DNA fragmentation, likely because the DNA was isolated from archival tissue samples (formalin-fixed paraffin-embedded tissues). The phylogenetic analysis of the acquired genomes showed that HPV16 DNA was relatively highly conserved, and the most divergent local sequences (≤24 substitutions) demonstrated less than 0,36% differences with the reference genome, as it had been described earlier [91]. A total of 93 non-redundant variants were observed across the local genomes, of which 25 had not been documented previously. Most variants were localized in non-coding regions and some protein-coding regions, with the most amino acid substitutions documented in the HPV capsid proteins L1 and L2. These were shown to be characteristic of the geographical region [92]. Several of the substitutions identified in the isolates from E1, E2, E5, and E6 oncoproteins had already been described as necessary in increased HPV16 pathogenicity [93,94,95], while no amino acid substitutions were identified in the E7 oncoprotein. This study, as two other by the Early-Career researchers, was done in the frame the project of the Latvian Science Council “Human papillomavirus genome associated correlates of disease progression and treatment response for cervical neoplasms and cancer” (LZP-2021/1-0484) shed light on the genomic variants of the most prevalent HR-HPV in Latvia–HPV16 and identified previously undescribed variations in the HPV16 genome.

The section was closed by the presentation “**Characteristics of the Oncoproteins E6 and E7 of HPV16 Isolates Circulating in Europe with Focus on the Baltic Region**” given by **Juris Jansons** (Institute of Microbiology and Virology, Riga Stradins University, Pathology Institute, and Biomedical Research and Study Centers, Riga, Latvia). This presentation continued on the topic of HPV16 genetic variability in Latvia, with a specific focus on the two oncoproteins E6 and E7. Similarly, using DNA isolated from archival cervical tissue samples, samples with high or medium HPV16 load were subjected to sequencing. In total, 32 sequences were retrieved. While E7 sequences remained conserved, multiple amino acid substitutions were revealed in E6, with the most common ones being R17 (11.4%), Q21 (3.7%), D32 (1.9%), H85 (4.6%), and L90 (48.7%). Interestingly, some of the substitutions were localized in the domains involved in p53-binding, and one was localized within the functional motif of E6. The study concluded that while the substitutions did not seem to affect the main functional domains of the E6 oncoprotein, substituted amino acid positions formed covariance networks indicative of viral evolution under selective pressure, well established for such RNA viruses as HCV and SARS-CoV-2 [96,97], yet largely unexplored for HR-HPVs.

### 4.3. HPV Infection & Cancer Surveys

The third section was chaired by Dr. Vanja Berggren (Karolinska Institutet, Stockholm, Sweden) and was opened by the plenary lecture “**HPV Infection: Present and Future of Diagnostic and Prognostic Surveys**” by Prof. **Ruth Tachezy** (Faculty of Science, Charles University, Prague, Czech Republic). Several diagnostic techniques are employed to detect HPV infections, each varying in sensitivity, specificity, positive and negative predictive value, and logistical feasibility. While HR-HPV testing as a means of primary screening and introduction of HPV vaccines was a great leap forward in mitigating HPV-related diseases, the field of biomarkers for patient stratification and disease prognosis is still actively studied. Prof. Tachezy discussed several types of potential biomarkers, such as circulating HPV-specific antibodies, circulating cell-free and tumor DNA, DNA methylation, extracellular vesicles among others. HPV-specific antibodies represent an indirect measure of HPV detection. For HPV antibody detection, several antigens can be used—VLPs (L1 & L2 proteins), which provide type-specific information, HPV pseudovirions, which are mainly used for neutralizing antibody detection, and HPV E6/E7 proteins, which are rarely found in the healthy population and have shown promise in disease prediction. Elevated hazard ratios have been demonstrated for HPV DNA and HPV oncoprotein-specific antibodies in oropharyngeal cancer, and HPV presence and integration status have been associated with survival [98]. Additionally, some results highlight the usefulness of HPV E6/E7 antibodies even more, which demonstrated that the presence of these antibodies can be detected up to 28 years before diagnosis of oropharyngeal cancer, while in the healthy population, they remain rare. Moving to other markers, Prof. Tachezy discussed miRNAs in HPV-related cancers. Their studies have demonstrated deregulated miRNA expression patterns in HPV-related cancers, yet there is considerable variation between studies, since several factors, such as the sampled material, seem to impact these patterns [99,100,101,102]. TME analysis also offers a source of biomarkers. Prof. Tachezy’s group demonstrated significant differences in immune cell populations found in the TME in both tumor parenchyma and stroma [103,104]. The last biomarker the lecture covered was circulating nucleic acids. Some studies have shown that after treatment, in a large portion of oropharyngeal cancer patients, circulating tumor HPV DNA disappears, while in some cases, increases have been demonstrated and are associated with disease recurrence and worse survival. Overall, these molecules seem to be very promising predictors of disease [105,106,107,108].

In the next talk, **Deborah Holzapfel** (Enzo Life Sciences, Farmingdale, NY, USA) described “**Detection of Human Papillomavirus (HPV) Nucleic Acid in FFPE Samples with AMPIVIEW^®^ RNA Probes, Powered by Enzo’s LoopRNA™ ISH Technology**”. Deborah Holzapfel represented Enzo Life Sciences and described one of the company’s newest products targeted at the detection of HPV nucleic acid in formalin-fixed tissues using the basis of in situ hybridization. Their new product AMPIVIEW^®^ RNA probes has been designed to detect both high and low-risk HPVs in tissue samples. The talk demonstrated that using the AMPIVIEW^®^ RNA probes in situ hybridization sensitivity matches PCR sensitivity during testing of tissues with low and high-grade lesions. Deborah Holzapfel also highlighted the potential benefits of such an HPV nucleic acid detection approach. Firstly, while PCR assays require the homogenization of samples, in situ hybridization results can be observed without disrupting the tissue morphology. And secondly, the expression and spatial localization within the tissues of the target genes can be easily observed and documented.

Last of the Early-Career researcher presentations on the conference “**High risk HPV positive cervical squamous cell carcinomas: correlation of tumor grades with virus load and expression of p16, p53 and Ki67**” was given by **Dr. Karina Biserova** (Pathology Institute, Pauls Stradins Clinical University Hospital, Riga, Latvia). Dr. Biserova is a certified pathologist, her talk began with an overview of the immunohistochemical evaluation of tissues–the technique and what pathologists look for to diagnose squamous cell carcinomas. She also overviews the markers analyzed in the reported study. Firstly, Ki-67 is a marker of cell proliferation, and stains nuclei of cells undergoing mitosis [109]. Second, p16 is a tumor suppressor protein whose tumor-suppressing actions are upregulated in the presence of HPV; thus, it serves as a surrogate marker of HPV-related processes [110]. Lastly, p53 is a tumor suppressor protein encoded by the TP53 gene, involved in several signalling pathways controlling cell division, cycling, and apoptosis [111]. The reported study combined HR-HPV genotyping with histochemical analysis and aimed to investigate the associations between cervical cancer severity and HR-HPV genotypes, multiplicity of infection, virus load, and expression of cell cycle and immune checkpoints and proliferation markers p16, p53, and Ki-67. Among 76 cervical cancer tissue samples, all were HR-HPV-positive, and HPV16 was the most prevalent genotype, followed by HPV33. All tumors were p16-positive with varying levels of p16 expression, overall increasing with increasing tumor grade. Similarly, the percentage of Ki67-expressing cells and Ki-67 signal intensity tended to increase with increasing cancer grade, and HR-HPV loads were shown to correlate with the percentage of Ki-67-positive cells, positively with HPV39 and negatively with HPV18 loads. Dr. Biserova noted that while there were associations observed, the staining was quite heterogeneous, and it may be more useful in evaluating cervical epithelial neoplasia. Aberrant p53 expression was detected in six cervical cancer cases, with most coming from patients older than 60 years and with grade 3 cancers. Interestingly, a positive correlation was observed between p53, cancer grade, and HPV18 load. The reported study concluded that high-grade cervical cancers can be characterized by high loads of HPV16 and HPV39, and a high level of p16 expression. Even though aberrant p53 expression correlated with the grade of cancer, only a small number of positive samples were found; thus, further study is needed to evaluate the usefulness of this staining.

The section was closed by the presentation “**Design and expression of HPV16 oncoproteins E6 and E7 for the dynamic analysis of the levels of specific serum antibodies in women with and without cervical lesions**” by **Dr. Maria Isaguliants** (Institute of Microbiology and Virology, Riga Stradins University, Riga, Latvia). Several studies have pointed to HPV oncoprotein-specific antibodies as potentially useful for patient stratification and as predictive markers [112,113,114]. The reported study designed the E6 and E7 proteins for use in HPV-specific antibody detection in prospectively followed-up women with and without cervical lesions. The HPV E6 and E7 amino acid sequences of Latvian patients were retrieved and used for expression in *E. coli* using pET-based vectors. Preliminary antibody analysis included 12 serum samples from both HR-HPV-positive and negative women. The initial steps included validation of the acquired HPV proteins. This was done by analyzing sera samples in plates coated with the in-house and commercially available E6 and E7 proteins. The in-house proteins were shown to behave just as the commercially available ones, as a high correlation in signals was observed between in-house and commercial proteins. Investigations of anti-E6 antibodies revealed significant differences between HR-HPV negative patients and controls, as well as between those patients who α9 species HR-HPV positive and negative. Additionally, the initial testing revealed that no E6/E7-specific IgA could be detected. Further analyses included samples from the same prospectively followed-up women as well as 21 women who had had previous cervical disease. We could observe that in the prospectively followed-up women, anti-E6 antibody end-point titers were significantly higher, while in anti-E7, no difference was observed. Interestingly, in the group of women with a history of cervical disease, a positive correlation between endpoint titers and years after initial diagnosis could be observed. Additionally, we observed that in those patients who developed cervical disease across the study period, increases in E6 antibody end-point titers could be observed.

### 4.4. Prophylactic and Therapeutic HPV Vaccines & Their Implementation

The fourth and final section of the session, chaired by Dr. Maria Isaguliants, was opened by the presentation “**Public and Patient Engagements to Improve Uptake of Cervical Screening and HPV Vaccination in Developing Countries–the Case of Uganda**” from **Dr. Edward Kumakech** and **Dr. Vanja Berggren** (Faculty of Nursing and Midwifery, Lira University Uganda, and Karolinska Institutet, Stockholm, Sweden, respectively). Cervical cancer is the most prevalent cancer and the leading cause of cancer-related deaths among Ugandan women. Despite the availability of HPV vaccination with Gardasil4 and cervical cancer screening, uptake of these preventive measures remains critically low in Uganda. In 2020, only 30% of eligible girls received the HPV vaccine, and by 2019, only 10% of eligible women participated in cervical cancer screening. The reported study investigated the factors influencing the low uptake of preventive health services to aid public and patient engagement and improve the cervical cancer screening and HPV vaccination coverage. The study found that healthcare professional recommendations, education about cervical cancer in schools, and participation in community health outreach events were positively associated with HPV vaccination uptake. Conversely, lower vaccine uptake was associated with exposure to rumors, such as claims that the HPV vaccine causes infertility. When investigating attitudes towards cervical cancer screening, primary or tertiary education, marital status, perceived severity of cervical cancer, and knowledge about the disease were associated with higher participation in cervical cancer screening. In contrast, lack of modern contraceptive method use, limited knowledge about cervical cancer, lack of health care professional recommendations, and male partner disapproval negatively impacted screening uptake. Additionally, participant interviews revealed that screening uptake was also negatively impacted by misinformation about cervical cancer and cancer screening procedures, fears about exposure regarding male healthcare professionals, as well as a lack of accessible screening facilities, limited healthcare provider training, and the high cost of the diagnostic tests. The reported study highlighted the need to not only inform these communities about the risks of disease and safety of preventive measures, but also the need for major investments in the healthcare system and professional education.

## 5. Early-Career Researchers Contest

A commission of five researchers from different countries, and in different categories, from the early career researchers to distinguished professors assessed all presentations by the early career researchers in six categories: 1. Originality of research goal/aims; 2. Study design/Planning of the experiments; 3. Quality of obtained data including graphical presentation; 4. Novelty and significance of the results; 5. Overall quality of presentation, presentation skills; 6. Quality of abstract-reflection of study design, experimental part, results, discussion. Evaluation was done by giving points from “1” (the lowest) to “5” (the highest), summarizing points across the categories, and dividing by the number of reviewers. Not all reviewers assessed all presentations, some were excluded from reviewing due to the conflict of interest. The 1st prize for the best presentation and voucher for publication in the Special Issue of “VACCINES” was awarded to Dr Nikita Zrelov for his presentation “**Complete genome analysis of the human papillomavirus type 16 (HPV16) isolates from cervical carcinomas in Latvia**”. The second with publication voucher was received by **Enkhtuul Batbold** (Lyon Cancer Research Center, and University of Lyon, Université Claude-Bernard, Lyon, France) for presentation “**CCL5 is upregulated and secreted by HDV independently of the IFN response**”. The third was received by **Omolara Baiyegunhi** (Africa Health Research Institute, Durban, South Africa) for presentation “**Viral replication capacity influences HIV-1-induced metabolic and cytokine reprogramming of T Cells**”. All winners received the monetary awards and conference award certificates. The competition was sponsored by the scientific journal VACCINES (MDPI).

Thus, thanks to the support from VACCINES (MDPI), early-career researchers got an opportunity to impress a panel of international experts with their research prowess and through promoted publications, attract attention to their research by the broad research community.

## 6. Conclusions and Remarks

Virus-associated cancers are major contributors to global morbidity and mortality, and researchers and clinicians worldwide are actively working towards a better understanding of virus and infection biology, identification of prognostic and predictive biomarkers, as well as new prophylactic and therapeutic approaches. The VIRCAN2024 conference gathered experts, clinicians, and scientists working on various oncogenic viruses, allowing them to exchange results and foster knowledge exchange.
